# A Comparison of Open Ventral Hernia Repair Risk Stratification Systems: A Call for Consensus

**DOI:** 10.3390/jcm13226692

**Published:** 2024-11-07

**Authors:** Tamás Talpai, Dumitru Sandu Râmboiu, Cătălin Alexandru Pîrvu, Stelian Pantea, Mircea Șelaru, Dan Cârțu, Silviu Daniel Preda, Ștefan Pătrașcu, Nicolae Dragoș Mărgăritescu, Marius Bică, Valeriu-Marin Șurlin

**Affiliations:** 1 Discipline of Surgical Emergencies, Department of Surgery II, Victor Babes University of Medicine and Pharmacy, 300041 Timisoara, Romania; talpai.tamas@umft.ro (T.T.); pirvu.catalin@umft.ro (C.A.P.); pantea.stelian@umft.ro (S.P.); selaru_mircea@yahoo.com (M.Ș.); 2 Doctoral School, Department of Surgery, University of Medicine and Pharmacy of Craiova, 200349 Craiova, Romania; 3 III Surgery Clinic of “Pius Brinzeu” County Emergency Clinical Hospital Timisoara, 300723 Timișoara, Romania; 4 Department of Surgery, University of Medicine and Pharmacy of Craiova, 200349 Craiova, Romania; dan.cartu@umfcv.ro.com (D.C.); sdpreda@gmail.com (S.D.P.); stefan.patrascu@umfcv.ro (Ș.P.); dragos.margaritescu@umfcv.ro (N.D.M.); mariusbk@yahoo.com (M.B.); vsurlin@gmail.com (V.-M.Ș.); 5 First Clinic of Surgery, Craiova Emergency Clinical County Hospital, 200642 Craiova, Romania

**Keywords:** ventral hernia repair, CeDAR, VHWG, SSO, occurrence, complications

## Abstract

**Background/Objectives**: Ventral hernia repair (VHR) is a common surgical intervention linked to specific surgical site complications. In such occurrences, the related morbidity is often substantial. Although known risk factors have long been recognized, their systematic inclusion in risk stratification systems lacks universal validation. This study evaluates the effectiveness and correspondence of three risk assessment tools—CeDAR, VHWG, and the modified VHWG—in predicting postoperative wound complications in VHR patients. Methods: We analyzed data from 203 patients who underwent VHR for incisional midline or lateral wall hernia across two surgical departments between 2019 and 2023. Each patient was scored using CeDAR, VHWG, and the modified VHWG systems. Outcomes were assessed based on surgical site occurrences (SSOs) such as seroma formation, wound infections, and recurrences. **Results**: The incidence of SSOs was 8.9%, with two recorded deaths (0.89%). CeDAR scores showed a statistically significant relationship with SSOs but failed to accurately predict complication rates across subgroups. The VHWG grading system effectively predicted higher complication rates for grades III and IV compared to grades I and II, though its modified version did not show significant predictive improvements. Secondary outcomes indicated a higher SSO rate in patients requiring posterior component separation (TAR) and those with larger hernia defects, though the differences were not statistically significant. Major preoperative risk factors, including smoking, diabetes, and obesity, did not show significant correlations with SSO rates in this study. **Conclusions**: Current risk estimation tools inadequately predict SSOs in VHR. Enhancing prediction accuracy will require incorporating both patient-specific and surgical factors, potentially through advanced algorithms and large-scale studies.

## 1. Introduction

Following abdominal surgery, one of the most common long-term causes of morbidity is represented by inadequate abdominal wall fascial healing, with the subsequent formation of an abdominal wall defect, also known as a ventral hernia [1]. Its effect on a patient’s quality of life is often significant, and the patient may even develop life-threatening complications due to incarceration [2]. The surgical management of ventral hernias has evolved considerably over the past decades, with a shift from primary suture repair to mesh-based reinforcement techniques that have demonstrated superior long-term outcomes [1]. However, the repair of complex ventral hernias, characterized by large defect size, loss of domain, and associated comorbidities, remains a formidable challenge for surgeons.

Surgical repair, in the elective or acute setting, is considered the only definitive treatment. Current surgical techniques exploit different layers of the abdominal wall to increase its capacity but also to reinforce its tensile strength using prosthetic meshes, though the risk of recurrence and associated complications remains a serious concern [3].

Ventral hernia repair (VHR) is a common surgical procedure, with an estimated 350,000 to 500,000 procedures performed annually in Europe and the United States [4]. Their incidence seems to be increasing, which may be linked to the rising prevalence of risk factors and the aging population, with an estimated rate of open abdominal procedures between 2 and 16% [5]. Numerous patient-related factors have been correlated with hernia formation; these include the size and grade of contamination of the initial procedure and patient comorbidities (smoking, diabetes, obesity) [5]. Wound complications following open VHR remain one of the main concerns, given their significant role in ventral hernia formation, but also recurrence. As such, multiple strategies have been attempted to mitigate their effect, including minimally invasive techniques, prehabilitation programs, and various preoperative procedures, such as preoperative progressive pneumoperitoneum or botulinum toxin type A injections [6,7]. Given the heightened complexity of these cases, which are associated with increased recurrence rates and postoperative complications, careful patient selection and risk stratification is critical to optimize surgical outcomes [8,9,10,11].

However, as mentioned by Fligor et al., current risk stratification systems, including the Ventral Hernia Working Group (VHWG) grading and its modified version (mVHWG) by Kanters et al. and the Carolinas Equation for Determining Associated Risk (CeDAR), have yet to be universally validated [8,12,13].

To address this need, the current study aims to comparatively evaluate the performance of these three risk stratification systems in predicting postoperative outcomes following open ventral hernia repair and provide recommendations for a consensus-driven approach to risk assessment.

## 2. Materials and Methods

For the current study, data from VHR patients from two surgical departments in Romania, the First Clinic of Surgery of Craiova Emergency Clinical County Hospital and the III Surgery Clinic of “Pius Brinzeu” County Emergency Clinical Hospital, Timisoara, recorded between 2019 and 2023 were collected and analyzed. Each patient received a grade/score based on the above stratification, with the results being compared with actual wound complication rates.

The Institutional Review Board of the participating hospital granted ethical approval for this study, ensuring compliance with the ethical standards outlined in the 1964 Helsinki Declaration and its subsequent amendments related to human research ethics. This study’s protocol was reviewed and approved, with each approval registered through the relevant Institutional Regional Board. Local ethics committee approval for this study was obtained (No. 261/27 November 2023).

### Data Collection

**Figure 1:**
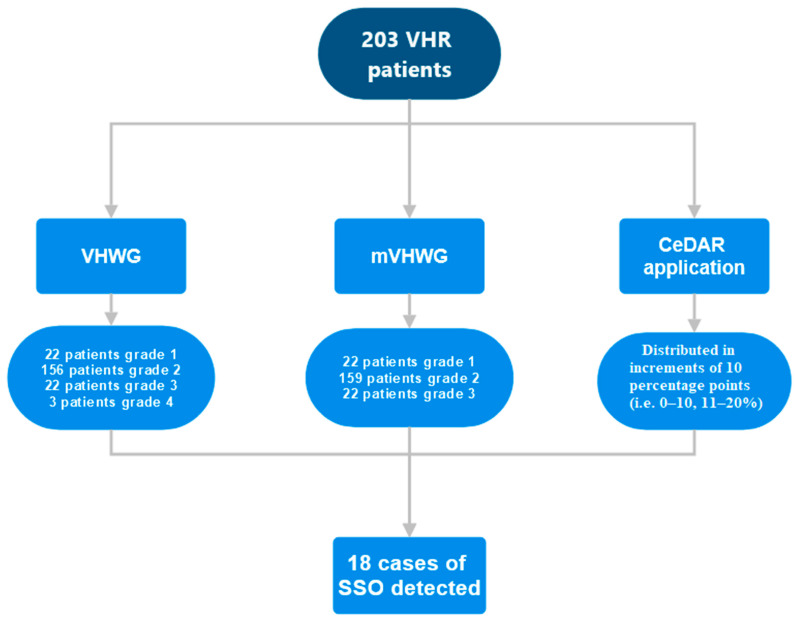
Flowchart with patient grading systems.

**Figure 2:**
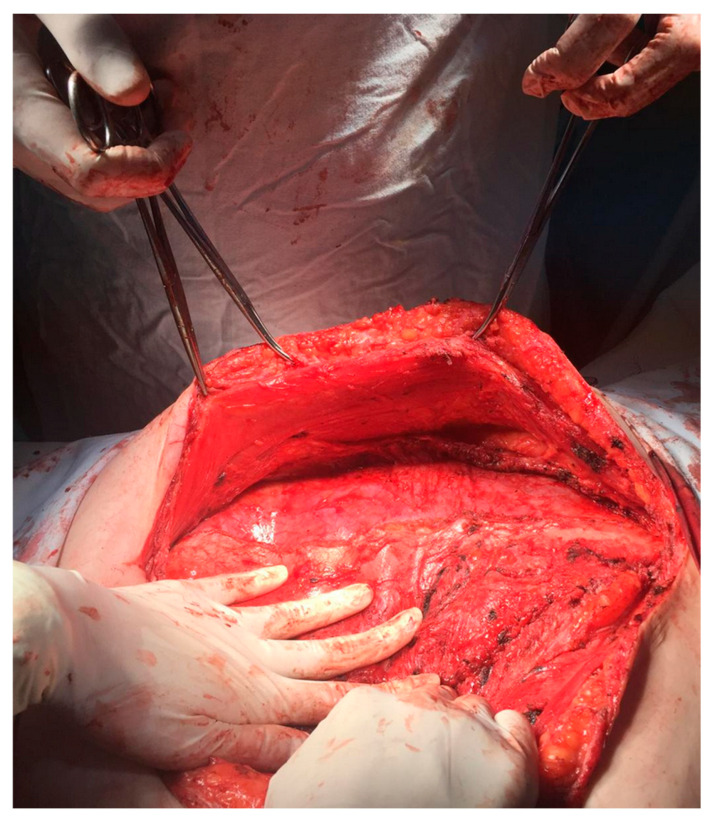
Unilateral transversus abdominis release (personal archive).

Patients who had either an incisional midline hernia repair or a lateral wall incisional hernia repair with the placement of a mid-weight polypropylene mesh in the retrorectus space were eligible for this study. The patients could also have had additional posterior component separation using transversus abdominis release (TAR), depending on the surgeon’s clinical judgment (Figure 1 and Figure 2).

Patient data were retrospectively extracted from their corresponding digital registries. Patient variables included general demographical information; presence of known systemic risk factors (active smoking, chronic obstructive pulmonary disease, diabetes, hypertension, ASA score, chemotherapy in the last year); defect size classification based upon the European Hernia Society recommendations (defect width marked as W1 if under 4 cm, W2 if between 4 and 10 cm and W3 if over 10 cm, respectively; M—midline localization of the defect, M1—subxiphoidal, M2—epigastric, M3—umbilical, M4—infraumbilical, M5—suprapubic), noting if the defect was confined to a single midline zone (M = 1) or to more than one (M > 1); the necessity to perform TAR; operative data; postoperative outcomes; and subsequent outpatient clinic visits [14].

The primary outcome was the incidence of surgical site occurrences (SSOs), defined as wound cellulitis, non-healing incisional wound, fascial disruption, skin or soft tissue ischemia, skin or soft tissue necrosis, wound serous or purulent drainage, stitch abscess, seroma, hematoma, and infected or exposed mesh in addition to SSI, seroma, wound dehiscence, enterocutaneous fistula, and the necessity of reoperation for such cases [15]. All SSOs were diagnosed clinically by their attending physician or via high-resolution computer tomography scans evaluated by a senior radiologist and described in their notes. A 30-day follow-up was also obtained through each center’s electronic registry. No long-term follow-up was obtained due to it being outside this study’s scope.

All operative protocols, standardized across institutions and years, specified an incision above the abdominal defect, lysis of the hernia sac, and intra-abdominal adherences, followed by retromuscular dissection of the posterior rectus sheath up to its lateral border. In cases of insufficient length to facilitate the posterior sheath closure or excessive tension, TAR was performed as per the surgeon’s clinical judgement. Posterior sheath closure was achieved using continuous sutures, but no information about the suture material used was consistently mentioned; thus, this parameter was excluded from this study. The anterior rectus sheath was sutured using permanent sutures.

All patients were scored using the CeDAR application and graded based on the VHWG scale and its modified version, developed by Kanter et al. [8,13] (Table 1).

CeDAR is a mobile application that analyzes a patient’s risk of developing wound complications following VHR and estimates the associated costs based on a risk assessment from data provided by the International Hernia Mesh Registry [11]. The application considers the following patient factors: untreated diabetes (defined as one of the following: blood sugar not monitored daily, average glycemia in the previous month over 180 mg/dL, or HbA1c exceeding 7.3%), tobacco use, previous hernia repair, pre-existing stoma, and body mass index (BMI). CeDAR also evaluates surgical factors such as concurrent gastrointestinal tract entry, abdominal infection, skin flap advancement, and component separation. The CeDAR app provides an estimated risk as a percentage and estimates of costs associated with potential wound complications (Table 2).

The VHWG grading was initially created to predict the risk of developing SSOs based on specific patient characteristics and level of wound contamination [13]. Grading defects range from 1, meaning a clean surgical field with no patient comorbidities, to grade 4, for cases of infected mesh or septic dehiscence. Moreover, its grading was intended to guide the choice of technique and prosthesis material (biological or synthetic). All incisional hernia patients were graded from 1 to 4.

The modified VHWG grading simplifies its initial format into three grades, given Kanters et al.’s observation that in their cohort, classifying patients with previous wound infections into grade 2 and gastrointestinal tract violation into grade 4 provided more accurate predictions of SSOs in their study [8].

Continuous data were described using means ± standard deviations (SDs), ranges (minimum–maximum), and medians (interquartile ranges). Categorical data were described using frequency and percentage. Continuous quantitative variables were analyzed via univariate analyses, and multivariate analyses were performed when a significant relationship was found between univariate ones. Data analysis was performed using GraphPad PRISM (ver. 10.1.1, 2023). The statistical significance threshold was established at the 0.05 level (2-sided).

Continuous data were described using means ± standard deviations (SD), ranges (minimum–maximum), and medians (IQRs, interquartile ranges). Categorical data were described using frequencies and percentages. Fisher’s Exact Test or the Chi-Square Test were used to evaluate the differences between groups of patients (nominal variables).

We performed logistic regression to assess the relationship between CeDAR scores, VHWG Grade, or modified VHWG Grade (predictor) and the likelihood of SSO (outcome, a binary yes/no outcome). Data analysis was performed using GraphPad PRISM (ver. 10.1.1, 2023) and SPSS (version 20, IBM). The statistical significance threshold was established at the 0.05 level (2-sided).

## 3. Results

A total of 203 patients met the inclusion criteria. The mean age (±SD) was 62.27 (±11.55) years, and the mean body mass index (±SD) was 30.4 kg/m^2^ (±range, 23.39–43.28 kg/m^2^). Of the patients, 32% were male. The main demographic results are shown in Table 3.

SSOs occurred in 18 patients (8.9%) and ranged from seromas or persistent drainage to mesh infection or fascial disruption. Among these cases, two deaths were recorded (0.89%).

Based on the determined CeDAR score, subgroups based on increments of 10 percentage points (i.e., 0–10%, 11–20%, etc.) were created, calculating the actual percentage of SSOs that occurred compared to the predicted value (Table 4). Although the latter subgroups did indeed develop SSOs more frequently, almost none of the subgroups correctly predicted the true complication rate. However, a significant relationship was found between CeDAR and SSOs (B = 0.058, *p* < 0.0001), suggesting a statistically significant small positive effect on the log-odds of SSOs for each one-unit increase in the CeDAR variable.

Similarly, patients were distributed in four subgroups based on their VHWG grading and the number of SSOs. Grades 1 and 2 were compared to grades 3 and 4. Twelve complications (6.7%) were observed for the grade 1 and grade 2 VHWG groups of patients, compared to the six complications (24%) observed in the grade 3 and grade 4 groups, which is significantly higher (*p*-value = 0.0128) (Table 5). We observed a statistically significant increase in the number of complications in grades 3 and 4 compared to grades 1 and 2 (24% vs. 6.7%, *p* = 0.0128). VHWG grades greater than 1 did not show statistically significant differences in SSOs compared to VHWG grade 1 (grade 2: B = 18.718, *p* = 0.998; grade 3: B = 19.699, *p* = 0.998; grade 4: B = 21.896, *p* = 0.998).

Patients were distributed in three subgroups based on their modified VHWG grading and the number of SSOs. Grade 1 was compared to grades 2 and 3. Although wound complications were more frequently observed in mVHWG grade 2 and 3 patients compared to grade 1, the difference was not statistically significant (11% vs. 0%, *p*-value = 0.23) (Table 6). Modified VHWG grades greater than 1 did not exhibit statistically significant differences in SSO compared to Modified VHWG grade 1 (grade 2: B = 18.697, *p* = 0.998; grade 3: B = 20.222, *p* = 0.998).

As for secondary outcomes, more SSOs were observed in patients with TAR than without TAR, but the difference was not statistically significant (17.6% vs. 8%, *p* = 0.38). Incisional hernias wider than 10 cm (EHS classification W3) had a higher incidence of wound complications (12.8%) compared to W1-2 patients (7.7%), but without statistical significance (12.8% vs. 7.7%, *p* = 0.28). Similarly, higher rates of SSOs were observed in patients that had lateral abdominal wall defects (12.2% vs. 8%, *p* = 0.371) or previous VHRs, without reaching statistical significance (27.8% vs. 16.2%, *p*= 0.205).

When evaluating the three major risk factors for incisional hernias, we did not find any significant difference between the groups: active smoking (*p* = 0.805), diabetes (*p* = 0.183), and obesity (*p* = 0.325).

Moreover, individually, none of the other risk factors included in the above-mentioned risk stratification systems (recent chemotherapy or radiotherapy (5.6% vs. 7.6%, *p* = 1.0); significant COPD or obstructive respiratory disease (27.8% vs. 15.7%, *p* = 0.193)) managed to reach statistical significance.

## 4. Discussion

Surgical site occurrences are a defined composite endpoint that covers seroma formation, hematoma, wound dehiscence, skin necrosis, formation of enterocutaneous fistula, and surgical site infection [15]. As shown in our results, the incidence of SSOs was 8.9%, which aligns with the results obtained by other authors [5,16,17,18]. An explanation for this wide variability may be the different terms used as endpoints in ventral hernia surgery and their diagnosis methods. Haskin et al. concluded that in the top 50 most cited ventral hernia publications from the past 20 years, only 18% used a standardized definition for each concept (SSI, SSO, surgical site occurrence requiring procedural intervention (SSOPI)) [19]. Such events can significantly increase hospital stays, costs, and patient morbidity. Juvany et al. have shown that SSOs can persistently predict the risk of hernia recurrence [16]. There is an increased need for standardized criteria that should be adopted across surgical practice in order to achieve consensus on both the treatments and outcomes that follow them.

Improving outcomes following VHR can be obtained by increasing SSO prediction accuracy through risk stratification systems or by the preoperative optimization of modifiable risk factors, such as smoking, diabetes, or obesity [10,20,21,22]. Numerous risk stratification systems fail to be externally validated or rely heavily on experts’ opinions. The three risk stratification systems evaluated did not manage to predict the occurrence of SSOs, similar to the results reported by Fligor et al. [12]. In fact, this study proves their weak predictive value in cohorts with a low incidence of SSOs.

Although the CeDAR application showed an increased SSO occurrence at higher percentages, it did not accurately predict their real occurrence rate in any incremental subgroup. Although it includes many significant patient comorbidities, hernia defect characteristics, such as defect size, loss of domain, abdominal wall localization, presence of previous mesh, and the use of bridging mesh or other surgical technique choices, are not evaluated using this score [17].

The VHWG grading scale is a four-tiered classification system used to assess the risk of complications following ventral hernia repair. It was developed in 2010 to provide a standardized approach to evaluating ventral hernia defects and guiding surgical decision-making. In the present study, the VHWG grading scale managed to predict a higher rate of SSOs in grades III and IV, compared to grades I and II, without managing to obtain similar results for the version modified by Kanters et al., who argued that some limitations of the original scale can be improved by reclassifying patients with previous wound infections into grade 2 while inserting gastrointestinal tract violation into the highest grade [8]. This resulted in a more accurate prediction of SSOs in their study. However, the SSO rate was not statistically significant when comparing patients in grade I against grades II and III.

The three major preoperative risk factors recognized for VHR are active smoking, obesity, and uncontrolled diabetes [16,17]. In our study, we could not find any statistically significant correlation (*p* = 0.805, 0.183, 0.325). These three can disrupt the normal healing process of the surgical incision, leading to potential SSOs in the short term or long-term hernia recurrence if interventions are not made. However, recent studies have challenged their actual clinical relevance and true impact on modification of clinical management. For example, Petro et al. observed that in 418 active smokers extracted from the Americas Hernia Society Quality Collaborative and propensity-matched to non-smokers, more SSOs were diagnosed (12.0% vs. 7.4%, *p* = 0.05), but without an increased surgical site occurrence rate requiring procedural interventions [22]. Similar results can be found when correlating diabetes with SSOs, given its described effect on promoting delayed wound healing, but with inconclusive results regarding an increased rate of SSOs requiring intervention in this subgroup of patients [19]. However, a remark must be made on the absence of differentiation between controlled and uncontrolled diabetes, based on fasting glucose levels above 180 mg/dL, HbA1C over 9%, or increased insulin dose needs, creating a potential confounder for this risk factor. Finally, obesity has been included in virtually every risk stratification system, conventionally using BMI over 30 kg/m^2^ as a cutoff value. Given the current worldwide increase in obesity prevalence, several authors have suggested creating a stratified risk model, dependent on BMI value, with a linear increase in postoperative SSOs, but also highlighting the fact that malnourished and underweight patients may present an even greater risk of SSOs than those with a BMI over 40 kg/m^2^ [15,20,21,22].

Moreover, increased BMI is associated with increased adipose tissue, potentially increasing operative time or the need for concomitant subcutaneous advancement flaps [23]. No BMI exclusion criteria were used in our study. Our cohort included patients in the 23.38–43.2 kg/m^2^ range. However, patients deemed to have potentially modifiable risk factors were counseled about this possibility.

Although not systematically introduced in our practice, preoperative prehabilitation attempts can be made to address these three risk factors. However, current results are relatively scarce or have been shown to improve outcomes only for those with BMI > 40 kg/m^2^ [24]. Among the three evaluated scores, only the CeDAR app takes into consideration the three major risk factors, with a possible utility in selecting surgical candidates for prehabilitation.

Other hernia-related risk factors, such as defect size and the necessity for TAR or lateral abdominal wall defects, have all shown an increased risk of SSOs without reaching statistical significance in our study [25,26]. Defect size, evaluated using the European Hernia Society classification and using the symbol M to express midline localization and W for defect width, was first used to assess whether a defect size greater than 10 cm in width (W3) increased the risk of SSOs compared to defects smaller than 10 cm in width (W1 and W2). This resulted in a higher rate of SSOs, but without statistical significance (12.8% vs. 7.7%, *p* = 0.28). None of the evaluated risk stratification systems included hernia size. However, it can be implied that the larger the defect, the more complex the VHR case becomes, increasing operative time, potential blood loss, and the need for TAR [3]. In our study, TAR was utilized, increasing the rate of SSOs to 17.6% compared to 8% for those that did not necessitate it. In a meta-analysis conducted by Vasavada et al., in a pooled analysis of 5284 patients who underwent TAR, the cumulated SSO rate was 21.72%, with age, sex, increased BMI, presence of comorbidities, defect size, smoking, and prior recurrence determined as risk factors [26]. Although usually indicated for defects larger than 10 cm in width, TAR may also be utilized in smaller defects, depending on the chronicity of the hernia and rectus muscle retraction [3]. A significant number of patients included in this study had non-midline ventral hernias (41/203, 20.2%). Articles describing large cohorts of open VHR for non-midline ventral hernias using a retromuscular approach are scarce. However, similar to Veyrie et al., SSO formation was more common in those patients compared to midline VHR cases [25].

The type of mesh used was not controlled for in this study due to the exclusive use of midweight polypropylene, which limits the ability of this study to draw definitive conclusions about its effects on SSO or early recurrence in VHR; however, Groene et al. found that the use of this mesh was correlated with the lowest rate of SSIs when compared to light- and heavyweight synthetic mesh usage [27].

Regarding the technique used in fascial closure, the majority of our operative reports did not specify these aspects, but the concept of closing the fascia without excessive tension was always respected. Suture material was also not specified, but a permanent suture was utilized as per each surgical team’s internal protocol. Fox et al.’s findings support these practices; they noted that fascial closure technique had no effect on SSO rates but found that using a resorbable suture for fascia increased SSO rates by 62% [28].

On a final note, creating a risk stratification system for predicting SSOs in VHR patients may prove a challenging endeavor, and validating it so it can be applied to as many patients as possible—all the more so. The estimation tools used in this study did not manage to predict SSOs in low-risk patients using the CeDAR application. Utilizing large cohorts and training deep learning algorithms on clinical and radiographical data to predict outcomes such as SSIs or SSOs could increase prediction accuracy in the future [29].

Our study’s potential limitations include the absence of a larger study group, given the low incidence of SSOs recorded in our study (18/203 SSOs). Larger differences could possibly be observed in greater cohorts through multi-center studies or by extracting data from renowned registry databases. Another source of potential bias may be the lack of representation of the two extremes of BMI, as well as the retrospective design employed.

## 5. Conclusions

In the present study, current risk stratification systems failed to accurately predict SSOs in low-risk patient cohorts. This study also demonstrated a significant relationship between SSOs and higher-risk groups using CeDAR and the VHWG grading system but failed to accurately predict the incidence of SSOs across low-risk subgroups. Higher rates of SSOs were also observed in more complex ventral hernias, as defined by the need for TAR or larger defects. The utility of the examined scores has been previously validated, adding clinical value to surgeons’ decision-making; however, their satisfactory predictive value in high-risk cohorts and prediction in low-risk patients can be improved. Patient-related factors are insufficient to predict surgical site outcomes, as shown in our findings; rather, an implementation of hernia-specific characteristics such as defect size, localization, and key aspects of surgical techniques is needed to create risk profile-specific care for ventral hernia patients.

## Figures and Tables

**Table 1:** Ventral Hernia Working Group scale and its modified version.

**Grade 1**	**Grade 2**	**Grade 3**	**Grade 4**
Low risk	Co-morbid	Potentially contaminated	Infected
-Low risk of complications -No history of wound infection	-Diabetes, COPD, immunosuppression, active smoker, obesity	-Previous wound infection -Intestinal stoma -Violation of GI tract	-Mesh infection -Septic dehiscence
**Grade 1**	**Grade 2**	**Grade 3**	
Low risk	Co-morbid	Contaminated	
-Low risk of complications -No history of wound infection	-Diabetes, COPD, immunosuppression, active smoker, obesity	-Intestinal stoma -Violation of GI tract -Infected mesh -Septic dehiscence	

COPD, chronic obstructive pulmonary disease; GI, gastrointestinal.

**Table 2:** Summary of factors evaluated in the CeDAR application.

	Patient-Related Factors	Surgical Technique-Related Factors
CeDAR application	-Uncontrolled diabetes (average glycemia in the previous month over 180 mg/dL or HbA1c exceeding 7.3%) -Use of tobacco products -Previous hernia repair -GI tract violation or stoma -Active abdominal infection -Height/weight	-Concomitant skin and subcutaneous advancement flaps -Concomitant component separation techniques

**Table 3:** Patients’ main demographic results.

Characteristics *	Patients (n = 203)
Age	
mean ± SD	62.27 ± 11.55
median (IQR)	63 (54–71)
range	27–65
Gender, n (%)	
Male	64 (31.5%)
Female	139 (68.5%)
BMI	
mean ± SD	30.4 ± 4.6
median (IQR)	29.8 (26.9–32)
range	23.4–43.3
ASA, n (%)	
1	10 (4.9%)
2	93 (45.8%)
3	93 (45.8%)
4	7 (3.4%)
HTN, n (%)	148 (72.9%)
Diabetes mellitus, n (%)	61 (30%)
Collagen disease, n (%)	2 (1%)
Tobacco use, n (%)	113 (55.7%)
COPD, n (%)	34 (16.7%)
Recent chemotherapy, n (%)	15 (7.4%)
Location, n (%)	
M = 1	95 (46.8%)
M > 1	108 (53.2%)
Lateral defect, n (%)	41 (20.2%)
Width, n (%)	
W1	24 (11.8%)
W2	132 (65%)
W3	47 (23.2%
TAR, n (%)	17 (8.4%)
VHWG, n (%)	
1	22 (10.8%)
2	156 (76.8%)
3	22 (10.8%)
4	3 (1.5%)
Modified VHWG, n (%)	
1	22 (10.8%)
2	159 (78.3%)
3	22 (10.8%)
CeDAR	
mean ± SD	21.79 ± 14.48
median (IQR)	17 (13–26)
range	7–85
SSO, yes, n (%)	18 (8.9%)

BMI—body mass index; ASA—American Association of Anesthesia; HTN—arterial hypertension; COPD—chronic obstructive pulmonary disease; TAR—transversus abdominis release; VHWG—Ventral Hernia Working Group; CeDAR—Carolinas Equation for Determining Associated Risk; SSO—surgical site occurrence. * Numbers are shown as numbers (percentages) or means ± standard deviations (SDs), medians (interquartile ranges, IQRs), and ranges.

**Table 4:** CeDAR-predicted risk.

CeDAR Predicted Risk	0–10%	11–20%	21–30%	31–40%	41–50%	51–60%	61–70%	71–80%	81–90%
Wound complications	1	6	2	3	0	2	0	2	2
Total patients	40	82	45	17	9	4	1	3	2
Wound complication rate (%)	2.5%	7.3%	4.4%	17.6%	0	50%	0	66.7%	100%

CeDAR—Carolinas Equation for Determining Associated Risk.

**Table 5:** Ventral Hernia Working Group complication rates.

Characteristics	Ventral Hernia Working Group (VHWG) Grades			
Wound complications, n (%)	Grade 1 (n = 22)	Grade 2 (n = 156)	Grade 3 (n = 22)	Grade 4 (n = 3)
	0	12 (7.7%)	4 (18.2%)	2 (66.7%)

**Table 6:** Modified Ventral Hernia Working Group complication rates.

Characteristics	Modified Ventral Hernia Working Group (VHWG) Grades		
Wound complications, n (%)	Grade 1 (n = 22)	Grade 2 (n = 159)	Grade 3 (n = 22)
	0	12 (7.7%)	4 (18.2%)

## Data Availability

Data are available on request from the authors.
